# Which factors lead to frequent attendance in the outpatient sector among individuals in the second half of life? Evidence from a population-based longitudinal study in Germany

**DOI:** 10.1186/s12913-018-3487-x

**Published:** 2018-08-30

**Authors:** André Hajek, Hans-Helmut König

**Affiliations:** Department of Health Economics and Health Services Research, Hamburg Center for Health Economics, University Medical Center Hamburg-Eppendorf, Martinistraße 52, 20246 Hamburg, Germany

**Keywords:** Primary health care, General practitioners, Health care utilization, Health services needs and demand, Primary care, Outpatient sector

## Abstract

**Background:**

Despite only constituting a small percentage of the population, frequent attenders place a tremendous burden on the healthcare system in Germany. Whilst there are some cross-sectional studies that examine the correlates of frequent attendance among older adults, there are only a few longitudinal studies that analyze the factors that lead to frequent attendance among middle-aged or older adults. Thus, the aim of this study was to investigate the factors leading to frequent attendance in the outpatient sector longitudinally.

**Methods:**

Data was drawn from three waves of a large, population-based sample of community-dwelling individuals aged 40 and above in Germany (*n* = 1049 in fixed effects regression). Individuals were classified as frequent attenders (GP visits) if they had, on average, visited a GP every second month in the previous 12 months. The same logic was applied for specialist visits.

**Results:**

Conditional FE logistic regressions showed that the onset of frequent attendance (GP visits) was negatively associated with age [OR: 0.91, 95% CI: 0.87–0.95], a change in employment status from employed to unemployed [OR: 2.26, 1.17–4.39], decreases in physical functioning [OR: 0.98, 0.97–0.99], worsening self-rated health [OR: 1.40, 1.11–1.78], and increases in physical illnesses [OR: 1.18, 1.06–1.32]. Similarly, the onset of frequent attendance (specialist visits) was associated with age [OR: 0.95, 0.92–0.98], decreases in physical functioning [OR: 0.99, 0.98–1.00], worsening self-rated health [OR: 1.50, 1.25–1.79], and increases in physical illnesses [OR: 1.24, 1.13–1.35].

**Conclusions:**

Need factors in particular were associated with the onset of frequent attendance. This relation did not vary by gender nor education, which may indicate that individuals only start to use health services more frequently when their needs increase.

**Electronic supplementary material:**

The online version of this article (10.1186/s12913-018-3487-x) contains supplementary material, which is available to authorized users.

## Background

A large share of general practitioner (GP) and specialist visits can be attributed to a small proportion of health insured individuals [1]. For example, it has been shown that the most frequent 1% of attenders accounted for 6% of all GP consultations (Leeds, four general practices; 44,146 patients and 470,712 consultations; year 1991 to 1995) [2]. Consequently, these so-called frequent attenders place a tremendous burden on the health care system [3], underpinning the significance of characterizing this group in detail. A recent systematic review identified some studies investigating the correlates of frequent attendance in late life [4]. Frequent attenders in late life, for example, were older and had more physical illnesses compared to non-frequent attenders [5]. Indeed, the association between physical illnesses and frequent attendance was found in six out of ten reviewed studies at the primary care level [4].

The systematic review concluded that much of the current knowledge on frequent attendance is based on cross-sectional studies, and noted a lack of longitudinal studies investigating the determinants of frequent attendance in later life [6–8]. As the number of individuals in old age is projected to increase considerably in the next few decades, it is key to examine the characteristics of this particular age bracket in further detail. According to the calculations of the Federal Statistics Office, 20 million individuals in Germany were aged 65 and over (whole population 80.8 million) in the year 2013 [9]. In the year 2060, it is projected that the number of individuals in Germany ≥65 years will increase to 33 million (whole population: 67.6 million) [9].

To date, one short-run (9 months) *longitudinal* study using two waves of data examined the psychosocial determinants of frequent attendance (> 17 GP consultations in 9 months), however this study was restricted to patients with heart failure in Germany (*n* = 310, average age: 72.9 years±9.0 years) [8]. In multiple logistic regressions, physical problems and living alone at baseline were associated with subsequent frequent attendance. This estimation strategy was applied in another longitudinal study that examined the determinants of persistent frequent attendance among middle-aged individuals in the Canberra region (Australia) [6]. However, previous studies have (mainly) used a static set of baseline characteristics to predict subsequent frequent attendance and therefore have not fully exploited the potential of longitudinal data, as changes within individuals over time have not been examined. Only one recent study used panel regression models to investigate the (time-varying) determinants of frequent attendance longitudinally [7]. Thus, the objective of this longitudinal study was to investigate the determinants of frequent attendance based on a nationally representative study using panel data methods (exploiting within-variation). This knowledge may help to reduce the burden on the health care system caused by frequent attendance.

Compared to cross-sectional regression models, certain panel regression models can identify causal effects (with certain restrictions) under weaker assumptions [10]. Changes within individuals over time can be analyzed. Moreover, the problem of unobserved heterogeneity, which is a main challenge in large survey studies, is mitigated. For example, bias caused by genetic differences between individuals (which is almost impossible to quantify in large survey studies) is not a problem when certain panel regression models are used [10].

Most studies [11–14] focusing on the determinants of health care use or frequent attendance base their analysis on the principles outlined in Andersen’s behavioral model [15]. Andersen’s behavioral model delineates the determinants of health care use into three categories, namely predisposing characteristics (e.g., sociodemographic variables), enabling resources (e.g., monetary resources), and need factors (e.g., self-rated health, physical illnesses or depression). Adjusting for need factors, previous studies have demonstrated that other factors, such as social isolation, are associated with frequent attendance [5, 16]. This might point to over- or misuse of these services [17]. Hence, it is important to know the factors leading to frequent attendance in the outpatient sector.

The German health care system is characterized by the compulsory nature of health insurance. Nine out of ten individuals are insured by social statutory health insurance (SHI) funds, and one out of ten individuals is insured by private health insurances (PHI) funds. Self-employed individuals, employees exceeding a certain income-threshold, and civil servants can opt for PHI. Most outpatient care expenses are covered by SHI and PHI. Individuals can use outpatient specialist physician services without referral from GPs, The waiting time for appointments with outpatient physicians is generally short [18]. Further details regarding the health care system in Germany can be found elsewhere [19].

## Methods

### Sample

Longitudinal data were derived from the second (2002), third (2008) and fourth (2011) wave of the nationally representative German Ageing Survey (“Deutscher Alterssurvey”, DEAS; starting in 1996 (*n* = 4838)) and funded by the Federal Ministry for Family Affairs, Senior Citizens, Women and Youth (BMFSFJ). Non-institutionalized adults aged 40 and over (“second half of life”) were interviewed face-to-face by trained staff. This interview covers, among other things, sociodemographic information. Following this interview, individuals are asked to answer a standardized questionnaire covering more personal topics (e.g., subjective well-being, loneliness and health).

In the second wave, 5194 individuals were interviewed (response rate: 38%). In the third wave, 8200 individuals were interviewed (response rate: 38%) and in the fourth wave, 4855 individuals were interviewed (response rate: 56%). According to Klaus et al. [20] 20,715 individuals aged 40 to 85 years at first interview took part in the DEAS study.

New samples were introduced in the second and third waves, whereas the fourth wave was a pure panel survey. Specifically, 1524 participants who had already been interviewed in 1996, were re-interviewed in the second wave. In the third wave, 1995 participants were re-interviewed and 6205 participants were first time participants. Neller [21] reported similar response rates for other large survey studies conducted in Germany. These moderate response rates for the DEAS study reflect a trend of decreasing participation rates in surveys in Germany.

Refusal to participate further in the study and health reasons were the main reasons for a lack of follow-up data. Klaus and colleagues have provided further details concerning the DEAS study elsewhere [20].

### Dependent variable

Health care use (frequency of GP or specialist visits) was recorded for the preceding 12 months. With respect to specialist visits, individuals gave information about their visits to internists; gynecologists; ophthalmologists; orthopedist; ear, nose, and throat specialists; neurologists; psychiatrists; dermatologists; urologists; and other specialists (open answer). The frequency of visits for each specialist and for GPs was reported by the individuals as “never”, “2–3 times,” “4–6 times,” “7–12 times,” or “more often” (open answer). These answers were recoded as “never” = 0; “once” = 1; “2–3 times” = 2.5; “4–6 times” = 5; “7–12 times” = 9.5; and “more often” = 13.

In this study, individuals were classified as frequent attenders when they met the following criterion (*main model*):

- Frequent attender (GP visits): ≥ 6 GP visits in the past year
- Frequent attender (specialist visits): ≥ 6 specialist visits in the past year

Thus, individuals were classified as frequent attenders (specialist visits) when they visited a specialist on average every second month. Otherwise, they were classified as non-frequent attenders. The same rule applied for GP visits.

### Independent variables

Independent variables were selected based on the Andersen behavioral model. With regards to predisposing factors, age, gender, marital status (married, living together with spouse, and others (married, living separated from spouse; divorced; widowed; never married), as well as occupational status (working; retired; other: not employed) were used. Moreover, the enabling resource of (log) household net income in Euro was used.

With regard to need factors, self-rated health (1 = very good, 2 = good, 3 = average, 4 = bad and 5 = very bad) was used. Moreover, the number of physical illnesses (no or yes) was measured (cardiac and circulatory diseases; bad circulation; joint, bone, spinal or back problems; respiratory problems, asthma, shortness of breath; stomach and intestinal problems; cancer; diabetes; gall bladder, liver or kidney problems; bladder problems; eye problems, vision impairments; ear problems, hearing problems) (ranging from 0 to 11 physical illnesses). Depression was quantified using the established Center for Epidemiological Studies Depression Scale (CES-D, 15 items, 0–45; depression = 1 if CES-D ≥ 18) [22]. Physical functioning was quantified using the subscale “Physical functioning” of the SF-36 [23], ranging from 0 (worst) to 100 (best).

### Statistical analysis

Bivariate comparisons of non-frequent attenders and frequent attenders were done using Chi^2^-tests and independent t-tests for categorical and continuous data respectively. Subsequently, conditional fixed effects (FE) logistic regressions were conducted. Generally, a conditional FE logistic regression can be written as [27]:

$$ P\Big({y}_{i1}0,{y}_{i2}=1\left|{y}_{i1}+{y}_{i2}=1\Big)\right.=\frac{\exp \left[{\beta}^{\prime}\left({x}_{i2}-{x}_{i1}\right)\right]}{1+\exp \left[{\beta}^{\prime}\left({x}_{i2}-{x}_{i1}\right)\right]} $$

In cross-sectional observational studies, unobserved heterogeneity including genetic disposition can heavily bias the estimates. This problem can be mitigated by using panel regression models [10]. With respect to the model assumptions, differences exist between widely used panel regression models. This is especially the case for the assumptions regarding the association between unobserved time-constant factors and independent variables. The consistency of random-effects regressions rests on the assumption that the independent variables are not associated with the time-constant factors in a systematic way, whereas FE regressions produce estimates that are consistent when this strong assumption is not fulfilled (when the exogeneity assumption holds) [10]. Conditional FE regressions were used in this study for this reason. Hausman-tests supported this choice (e.g., Hausman test statistic was: Chi^2^(9) = 97.46, *p* < .001; main model with GP visits as outcome measure (Table 2, first column)).

Conditional FE regressions solely exploit changes within individuals over time (e.g., changes from not being a frequent attender to being a frequent attender over time). Time-constant unobservable factors are eliminated by the within-transformation. Consequently, solely factors varying within individuals over time can be used in conventional FE regressions. Therefore, it is worth emphasizing that FE estimates solely use information from individuals who have changes in frequent attendance (non-frequent attendance to frequent attendance or vice versa) from 2002 to 2011. For this reason, the findings can only be generalized to individuals in the population who changed their status of frequent attendance (so-called average treatment effect on the treated). As already argued by Brüderl and Ludwig [10], this is not a deficiency in the estimation strategy. This mirrors the fact that only some individuals changed their frequent attendance status over time. Further details are provided elsewhere [27].

In additional analysis, we also adjusted for cognitive function using the Digit Symbol Test (DST), which was adapted from the Symbol Substitution Test (DSST) [24], a widely used measure with a good reliability [25]. The DST measures motor speed, and processing speed of visual perception and information. Higher values reflect better cognitive function (ranging from 1 to 92).

Moreover, it was tested whether loneliness affects frequent attendance (by adding this factor to our main regression model). It was also tested whether the time-constant variables gender and education (quantified using the International Standard Classification of Education, ISCED-97 [26], with three categories: low (0–2), medium (3–4), and high (5–6)) moderate the association between need factors and frequent attendance. In further additional analysis, it was tested whether the age effect is non-linear (by adding squared and cubic age terms).

The criterion for statistical significance was set at *p* < .05. All analyses were conducted using Stata 15.1 (StataCorp, College Station, Texas, USA).

## Results

### Sample characteristics and bivariate associations

Sample characteristics for the observations included in FE regression analysis are displayed in Table 1 (pooled) and separated by status (non-frequent attenders vs. frequent attenders). Among these individuals (FE regression with frequent attendance (GP visits) as outcome measure), 51.6% were female. Mean GP visits were 6.7 (±3.5) in the total sample. Mean specialist visits were 6.4 (±5.1) in the total sample. Compared to non-frequent attenders (both, GP visits and specialist visits), frequent attenders had more physical illnesses, lesser physical functioning, and poorer self-rated health in the total sample.

**Table 1:** Sample characteristics, by status (non-frequent attenders vs. frequent attenders), wave 2 (2002) to wave 4 (2011)

Variables	GP visits			Specialist visits		
	Non-frequent attenders (*n* = 541)	Frequent attenders (*n* = 508)	*p*-value	Non-frequent attenders (*n* = 947)	Frequent attenders (*n* = 915)	*p*-value
Age in years: Mean (SD)	66.9 (10.6)	66.3 (65.4)	0.42	64.0 (10.9)	64.0 (10.9)	0.93
Married, living together with spouse (Ref.: Others): N (%)	373 (51.4%)	353 (48.6%)	0.85	704 (50.8%)	681 (49.2%)	0.97
Employment status: N (%)			0.05			0.34
- Working	125 (53.2%)	110 (46.8%)		312 (52.1%)	287 (47.9%)	
- Retired	368 (52.8%)	329 (47.2%)		534 (51.1%)	511 (48.9%)	
- Not employed	48 (41.0%)	69 (59.0%)		101 (46.3%)	117 (53.7%)	
Household net income in Euro	2443.9 (1902.5)	2507.7 (2585.4)	0.65	2690.7 (3528.8)	2681.9 (1700.0)	0.95
Number of physical illnesses: Mean (SD); Range	3.1 (2.0)	3.4 (1.9)	< 0.05	2.5 (1.7)	2.8 (1.7)	< 0.001
Physical functioning (from 0 = worst to 100 = best)	79.3 (23.1)	74.2 (25.2)	< 0.001	83.2 (21.7)	79.7 (23.1)	< 0.001
Self-rated health (from 1 = very good to 5 = very bad)	2.7 (0.8)	2.9 (0.8)	< 0.001	2.5 (0.8)	2.6 (0.8)	< 0.001
Absence of depression (CES-D ≥ 18)	501 (52.5%)	453 (47.5%)	0.05	887 (51.3%)	842 (48.7%)	0.17
Cognitive function (higher values reflect better cognitive function, ranging from 1 to 92)	41.6 (13.7)	40.5 (14.4)	0.25	43.7 (14.0)	43.9 (13.6)	0.86
Loneliness: Mean (SD)	1.7 (0.5)	1.7 (0.5)	0.18	1.7 (0.5)	1.7 (0.6)	0.40

*N* number, *SD* standard deviation, Comparisons between the two groups were done using t-test and chi-square procedures

### Regression analysis

The determinants of frequent attendance are displayed in Table 2 (main model). Pseudo R^2^ was .086 (GP visits as outcome measure) and .064 (with specialist visits as outcome measure), respectively. The number of observations was 1049 (with GP visits as outcome measure) and 1862 (with specialist visits as outcome measure). Total observations differ between frequent attendance (i) with GP visits as outcome measure and (ii) with specialist visits as outcome measure, as there were varying number of changes over time in these outcome measures.

**Table 2:** Determinants of frequent attenders (0 = Non-frequent attenders; 1 = Frequent attenders; cut-off: 6 GP or 6 specialist visits). Results of conditional FE logistic regressions. From wave 2 (2002) to wave 4 (2011)

Independent variables	(1)	(2)
	Frequent attendance (GP visits)	Frequent attendance (specialist visits)
Age	0.91***	0.95***
	(0.87–0.95)	(0.92–0.98)
Marital status: - other (divorced, widowed, single, married, living separated from spouse; Ref.: married, living together with spouse)	1.06	0.96
	(0.46–2.43)	(0.52–1.78)
Employment status: - retired (Ref.: employed)	1.81+	1.16
	(0.92–3.57)	(0.74–1.83)
- other: not employed	2.26*	1.31
	(1.17–4.39)	(0.84–2.04)
Log household net income	1.03	1.39+
	(0.62–1.72)	(0.96–2.02)
Number of physical illnesses (from 0 to 11)	1.18**	1.24***
	(1.06–1.32)	(1.13–1.35)
Physical functioning (from 0 = worst to 100 = best)	0.98***	0.99**
	(0.97–0.99)	(0.98–1.00)
Self-rated health (from 1 = very good to 5 = very bad)	1.40**	1.50***
	(1.11–1.78)	(1.25–1.79)
Depression (CES-D ≥ 18; Ref.: absence of depression)	1.25	1.06
	(0.71–2.22)	(0.70–1.62)
Observations	1049	1862
Individuals	476	836
Pseudo R^2^	0.086	0.064

Odds Ratios (OR) were reported; 95% CI in parentheses; *** *p* < 0.001, ** *p* < 0.01, * *p* < 0.05, + *p* < 0.10

Odds ratios were given (with 95% CI in parentheses). We give two examples to aid the reader who may be unfamiliar with the interpretation of conditional FE regressions. The OR for ‘non-employment’ (Ref.: employed) was 2.26 (95%-CI: 1.17–4.39; with GP visits as outcome measure)). This means that if a participant changes from being employed to non-employed, their odds of being a frequent attender are multiplied by 2.26 (ceteris paribus). Another example: The OR for the continuous variable physical illnesses is 1.18 (1.06–1.32; with GP visits as outcome measure). A one-unit increase in the number of physical illnesses increases the odds of being a frequent attender by 18%.

With respect to predisposing characteristics and enabling factors, conditional FE logistic regressions showed that the onset of frequent attendance (GP visits) was negatively associated with age [OR: 0.91, 95% CI: 0.87–0.95], Furthermore, changes from employed to non-employed were significantly associated with GP visits [OR: 2.26, 1.17–4.39] – as already mentioned in the first example. With respect to needs factors, the onset of frequent attendance (GP visits) was negatively associated with decreases in physical functioning [OR: 0.98, 0.97–0.99], worsening self-rated health [OR: 1.40, 1.11–1.78] and increases in physical illnesses [OR: 1.18, 1.06–1.32] – as already mentioned in the second example.

Similarly, with respect to predisposing characteristics and enabling factors, the onset of frequent attendance (specialist visits) was associated with age [OR: 0.95, 0.92–0.98]. With respect to need factors, the onset of frequent attendance (specialist visits) was associated with decreases in physical functioning [OR: 0.99, 0.98–1.00], worsening self-rated health [OR: 1.50, 1.25–1.79], and increases in physical illnesses [OR: 1.24, 1.13–1.35].

In additional analysis (see Additional file 1), the model was also adjusted for cognitive function and loneliness. However, in terms of significance, these models revealed similar results compared to our main model. Neither cognitive function nor loneliness were associated with the outcome measures.

We also tested whether the age effect was non-linear. To this end, quadratic as well as cubic age terms were included in the regression model. However, non-linear age effects were not detected. In further sensitivity analysis, we also checked whether the need factors were moderated by (i) sex and (ii) educational level. However, none of the interaction terms achieved statistical significance.

## Discussion

Based on a nationally representative sample among community-dwelling individuals ≥40 years, the aim of this study was to examine the determinants of frequent attendance in the outpatient sector longitudinally, using panel data methods. In conditional FE logistic regressions, an increase in need-factors (self-rated health, functional decline, and physical illnesses) was associated with the onset of frequent attendance. Furthermore, ageing was negatively associated with the onset of frequent attendance.

What does this study add to the current knowledge? Using a sample that is generalizable to the non-institutionalized population in the second half of life in Germany, our findings extend previous knowledge about the correlates of frequent attendance that is based on cross-sectional studies. Moreover, changes within individuals over time were investigated in this longitudinal study.

### Predisposing characteristics and enabling factors

First, it is worth emphasizing that our findings are generally difficult to compare with previous studies due to differences in the time horizon (cross-sectional vs. longitudinal study) and differences in statistical analysis (cross-sectional regression models vs. regression models dealing with panel data). This should be kept in mind when interpreting our results below. Moreover, with respect to our sensitivity analysis (frequent attendance, GP visits), these results appear unreliable (in terms of significance), and may suffer from inadequate statistical power.

The loss of a spouse can cause adverse health outcomes (e.g., increased depressive symptoms). However, it appears plausible that changes in marital status were not associated with the onset of frequent attendance in our study, as it had been adjusted for various need factors in the regression analysis. After adjusting for several need factors, frequent attendance was *negatively* associated with age in our study. This might be explained by the fact that with increasing age, individuals might become more pessimistic about the treatment of his or her ailments or chronic conditions. Furthermore, the perceived opportunity costs of physician visits, may increase with age. As for age and marital status, the evidence is mixed [4]. While there is some evidence showing a positive association between frequent attendance and older age, as well as living alone, other studies show that these factors are not associated with frequent attendance [4]. Differences in the results of our study can be mainly explained by differences in time horizon.

It has been shown that a job loss can change the health behavior of individuals [28]. This might explain why the onset of frequent attendance (GP visits) was associated with changes from employment to non-employment in our study.

With respect to enabling factors, it appears plausible that these factors are not associated with frequent attendance in our study because most of the health care services are free of charge in Germany. Moreover, co-payments are quite low in Germany. However, an association between lower income and frequent attendance has been recently reported in a cross-sectional study [5]. The differences between our study and the cross-sectional study could be explained by differences in the study design (cross-sectional vs. longitudinal study).

In total, our findings demonstrate that the onset of frequent attendance is neither associated with predisposing characteristics nor enabling factors longitudinally.

### Need factors

In this study, the onset of frequent attendance was associated with an increase in need factors. It is worth noting that the relation between the need factors and frequent attendance did not vary by gender and educational level in our study. The relationship between need factors and frequent attendance is plausible. The onset of need factors implies the recognition by the individual of symptoms or signs of an illness, which the individual then seeks to be verified and treated by a GP or specialist. In particular, chronic conditions can cause increased doctor visits [29, 30]. Future research is required to clarify the specific physical illnesses that can cause long-lasting frequent attendance.

This longitudinal study adds to the current knowledge, which is predominantly based on cross-sectional studies. For example, previous studies have shown that need factors such as the number of somatic diseases is associated with frequent attendance [4]. Two cross-sectional studies found a positive association between depression and frequent attendance [31, 32]. However, depression became insignificant in multiple regression analysis in previous research [8, 32]. Future research is required regarding the relation between the onset of frequent attendance and the occurrence of depression in the *inpatient* sector. Using FE regressions, Pymont and Butterworth also found that depression was not associated with the onset of frequent attendance in Australia (Canberra Region) [7]. They found that the onset of pain was associated with the onset of frequent attendance. In the study conducted by Pymont and Butterworth [7] it also becomes evident that FE regressions (which solely rely on changes within individuals over time) can yield very different results compared to cross-sectional regressions.

### Strengths and limitations

The use of a nationally representative study of non-institutionalized individuals ≥40 years is a major strength of this study. Changes within individuals over time were also analyzed. In addition, FE regressions were used reducing the problem of unobserved heterogeneity, which is a key challenge in large survey studies. Based on the Andersen model, various key factors were included in our regression model. Validated scales (e.g., CES-D) were used to quantify these factors. As self-reported outpatient visits in the past 12 months were used, the possibility of a recall-bias cannot be dismissed in this study. Nevertheless, this bias is assumed to be rather small [33]. While a sample selection bias cannot be ruled out, it has also been estimated to be rather small [34]. In order to mitigate the effect of sample selection bias, intense efforts were undertaken to increase response rate by the DEAS. For example, incentives were increased. Since 2008, €10 have been paid to respondents. Finally, the results of our study might not be generalizable to individuals residing in institutional settings.

## Conclusions

Our findings showed that the onset of frequent attendance is neither associated with most of the predisposing characteristics nor enabling factors, when using a longitudinal approach. However, need-factors (physical functioning, self-rated health, physical illnesses) were associated with the onset of frequent attendance in our study. This relation did not vary by gender nor education. This may indicate that individuals start to use health services frequently once their health needs increase. Frequent outpatient visits cause high health care costs [35]. The findings of this study may help to delay or avoid the onset of frequent attendance among older adults, as at least some of these conditions are potentially preventable or curable.

Future studies based on long running (e.g., 20 years and over) nationally representative panel studies may help to identify the determinants leading to persistent frequent attendance using panel data methods. Such studies would be of use as persistent frequent attenders are costly for healthcare systems [36].

## Additional file

Additional file 1: Determinants of frequent attenders (0 = Non-frequent attenders; 1 = Frequent attenders; cut-off: 6 GP or 6 specialist visits). Results of conditional FE logistic regressions. From wave 2 (2002) to wave 4 (2011). Sensitivity analysis. (DOC 71 kb)

## Abbreviations

BMFSFJ: Federal Ministry for Family Affairs Senior Citizens Women and Youth
CES-D: Center for Epidemiological Studies Depression Scale
DEAS: German Ageing Survey
DSST: Digit Symbol Substitution Test
DST: Digit Symbol Test
FE: fixed effects
GP: general practitioner
ISCED: International Standard Classification of Education
OECD: Organization for Economic Co-Operation and Development
PHI: private health insurances
SHI: statutory health insurance
