# Supplementary material for: Which factors lead to frequent attendance in the outpatient sector among individuals in the second half of life? Evidence from a population-based longitudinal study in Germany
Source: BMC Health Serv Res. 2018 Aug 30;18:673. doi: 10.1186/s12913-018-3487-x (PMC6117977; doi:10.1186/s12913-018-3487-x)
Supplement: Supplementary file 1 — Determinants of frequent attenders (0 = Non-frequent attenders; 1 = Frequent attenders; cut-off: 6 GP or 6 specialist visits). Results of conditional FE logistic regressions. From wave 2 (2002) to wave 4 (2011). Sensitivity analysis. (DOC 71 kb) [file 12913_2018_3487_MOESM1_ESM.doc]

Table S1. Determinants of frequent attenders (0 = Non-frequent attenders; 1 = Frequent attenders; cut-off: 6 GP or 6 specialist visits). Results of conditional FE logistic regressions. From wave 2 (2002) to wave 4 (2011). Sensitivity analysis. 
	(1)	(2)	
Independent variables	Frequent attendance (GP visits)	Frequent attendance (specialist visits)	
			
Age	0.91***	0.95**	
	(0.86 - 0.95)	(0.92 - 0.98)	
Marital status: - other (divorced, widowed, single, married, living separated from spouse; Ref.: married, living together with spouse)	1.95	0.84	
	(0.68 - 5.57)	(0.39 - 1.82)	
Employment status: - retired (Ref.: employed)	2.24*	1.14	
	(1.02 - 4.93)	(0.67 - 1.96)	
- other: not employed	2.15*	1.06	
	(1.01 - 4.56)	(0.63 - 1.77)	
Log household net income	1.53	1.24	
	(0.80 - 2.92)	(0.80 - 1.93)	
Number of physical illnesses (from 0 to 11)	1.15*	1.27***	
	(1.01 - 1.32)	(1.14 - 1.41)	
Physical functioning (from 0 = worst to 100 = best)	0.98**	0.99*	
	(0.97 - 0.99)	(0.98 - 1.00)	
Self-rated health (from 1 = very good to 5 = very bad)	1.35*	1.62***	
	(1.02 - 1.80)	(1.32 - 2.00)	
Depression (CES-D ≥ 18; Ref.: absence of depression)	1.27	0.90	
	(0.67 - 2.43)	(0.55 - 1.47)	
Cognition (Digit Symbol Test)	1.00	1.01+	
	(0.98 - 1.02)	(1.00 - 1.02)	
Loneliness (according to Gierveld and van Tilburg)	0.93	1.02	
	(0.62 - 1.40)	(0.74 - 1.39)	
			
Observations	738	1,388	
Pseudo R²	0.077	0.068	
Odds Ratios (OR) were reported; 95% CI in parentheses; *** p<0.001, ** p<0.01, * p<0.05, + p<0.10
